# Supplementary material for: Inefficient prioritization of task-relevant attributes during instrumental information demand
Source: Nat Commun. 2023 Jun 1;14:3174. doi: 10.1038/s41467-023-38821-x (PMC10235048; doi:10.1038/s41467-023-38821-x)
Supplement: Supplementary file 1 — Supplementary Information [file 41467_2023_38821_MOESM1_ESM.pdf]

Supplementary Discussion

Supplementary Table 1

Points missed per trial

	EV Sensitive	Random	Uncertainty Only
Estimate Naive	-79.89 (±1.77)	-117.89 (±1.50)	-23.19 (±0.41)
Estimate Consider PC	-36.71 (±1.74)	-23.79 (±4.72)	-4.61 (±0.49)
Intervene Naive	-14.12 (±0.25)	-21.65 (±0.26)	-2.04 (±0.024)

Supplementary Table 1. Points missed per trial Values show the expected missed earnings (EME) calculated as described below, with each entry showing means and SEM over participants in the respective task and strategy group. More negative values indicate more missed earnings.

Supplementary Table 2

PC (when hiVar revealed)

	EV Sensitive	Random	Uncertainty Only
Estimate	0.83 (±0.012)	0.67 (±0.020)	0.98 (±0.006)
Intervene	0.94 (±0.007)	0.65 (±0.028)	0.98 (±0.009)

Supplementary Table 2. Percent correct (PC) for the instrumental decision after revealing hiVar information.

Although participants did not receive trial by trial feedback, they could estimate the earnings they would miss by sampling the loVar relative to the hiVar lottery using the task description alone. **Supplementary Table 1** shows the earnings that participants could expect to miss given their sampling policies in each task.

In the Estimate task, participants knew that the sum of the draws had a 50:50 prior probability of being above or below the criterion, and that an observation from the loVar lottery would not alter this prior while an observation from the hiVar lottery would reduce the uncertainty. Thus, they could expect that the probability correct (PC) for the estimation decision would be 0.5 if they sampled the loVar lottery and a higher value, PC_{hivar}, if they sampled the hiVar lottery. For each trial in which they sampled the loVar lottery, therefore, participants could expect to miss a fraction of the points they would have received had they sampled the hiVar lottery equal to $\frac{0.5 - PC_{hiVar}}{PC_{hiVar}}$. To calculate the expected value of the loss due to sampling (expected missed earnings, EME) we then multiplied this trialwise value by the participant's probability of sampling the loVar lottery expressed as a fraction – (1 - %reveal hiVar x 0.01) – and finally,

by the maximum trial value of 500 points, to obtain EME = [(1 - %reveal hiVar x 0.01) x 500 x $\frac{0.5 - PC_{hiVar}}{PC_{hiVar}}$]. We also considered that participants could have estimated PC_{hivar} in two ways. They could have assumed that they would make a perfect guess given hiVar information, or they could estimate their actual PC_{hivar} (perhaps based on their confidence in their guesses; measured PC_{hivar} values are listed in **Supplementary Table 2**). The two rows for the Estimate task refer to these two scenarios, where PC_{hivar} is set to 1 for "Naive" and to the participant's actual PC_{hivar} for "Consider PC".

For either assumption, the average EME was several-fold larger for participants with EV-sensitive relative to Uncertainty-only strategies (Naïve: $p < 0.001$, Consider PC: $p < 0.001$, Mann-Whitney rank sum test, $n = 358$ vs 123 participants). Note that, under "Consider PC", EV-sensitive participants also had larger EME relative to those with Random strategies. This is because the latter participants had smaller PC_{hivar}, indicating that they did not make good use of the information they sampled (**Supplementary Table 2**). These participants thus lost points because of inaccurate guesses but did not lose many additional points by sampling a loVar lottery.

In the Intervene task, if participants sampled a lottery and observed a low draw they could exchange it for the average value of the lottery – that is, they could recuperate 15 points if they sampled the loVar lottery and 60 points if they sampled the hiVar lottery. Thus, EME are equal to this difference multiplied by the probability of sampling the loVar lottery (adjusted to reflect the fact that low draws happen on only half the trials), i.e., EME = (1-%reveal hiVar x 0.01) x (15-60)/2.

Here as well, the average EME was 7-fold larger for participants with EV-sensitive relative to Uncertainty-only strategies ($p < 0.001$, Mann-Whitney rank sum test, $n = 402$ vs 109 participants). Note that in this task the post-sampling decisions were trivial and participants with non-random strategies almost always performed them correctly (**Supplementary Table 2**). For simplicity, we assumed that PC_{hivar} is equal to 1 for all groups (although note that this overestimated the losses due specifically to sampling for the Random group that had lower PC).

Supplementary Figure 1 (Fig. S1)

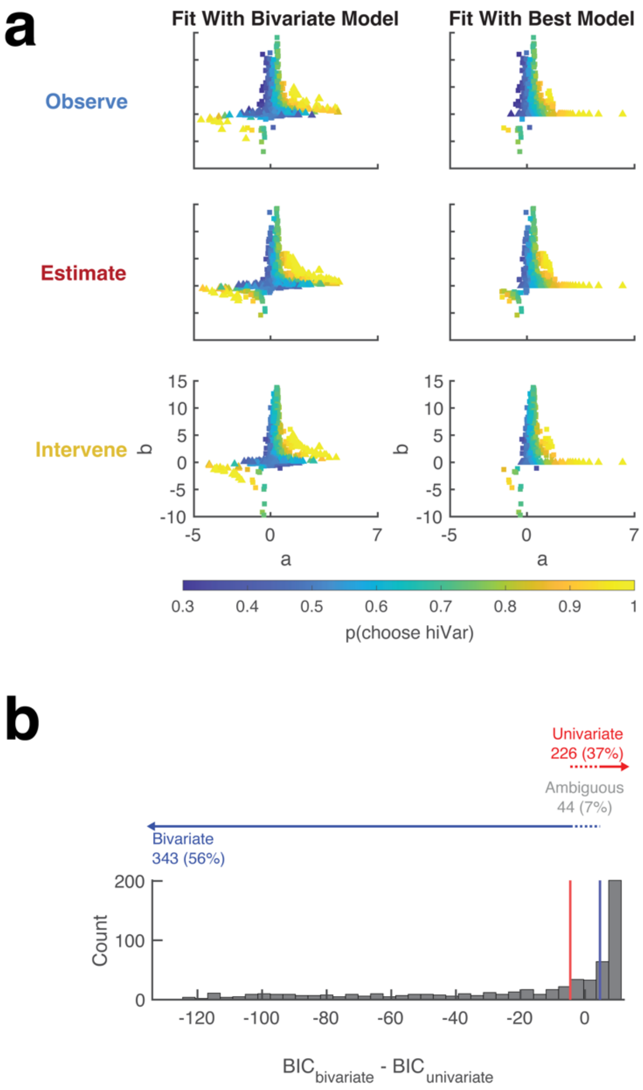

Fig. S1. Modelling sampling decisions (a) Scatterplots of fitted parameters (slope, y-axis versus intercepts, x axis) under two alternative methods. Each point is one participant and color indicates %reveal hiVar. In the left column, all participants are fit with a bivariate model; in the right, each participant is fit with the best-fitting of a bivariate or univariate model (see *Methods*). Circles indicate participants who were best fit by the bivariate model (EV-sensitive) and triangles indicate those who were best fit by the univariate model (EV-insensitive). The sizable group of points with negative intercepts is prominent in the left column but largely absent in the right column. **(b) Differences in BIC scores in the Estimate task.** The vertical lines show two criteria whereby participants are classified as EV-sensitive by default (blue) or as EV-insensitive by default (red). The horizontal red and blue arrows and labels show the number (%) of participants assigned to each class according to the corresponding criterion. Only 7% of participants were inconsistently classified (“Ambiguous”, dashed portions).

Supplementary Figure 2 (Fig. S2)

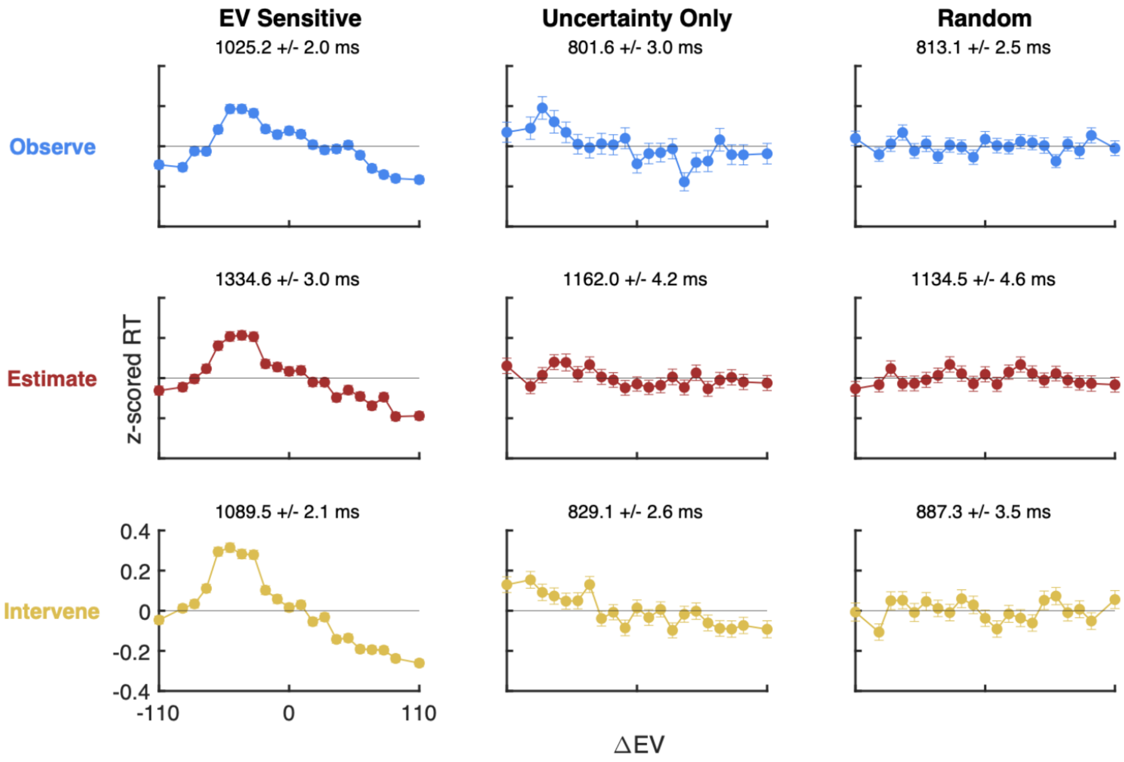

Fig. S3. Reaction Times (RT) to make the initial reveal decision. For each participant, RTs were z-scored within each block and averaged across blocks. The points show mean and standard errors across n = 610 participants; some error bars are smaller than the points. The values above each panel show the average raw RT across all ΔEV values in that condition. Units and scaling are indicated in the bottom left panel and are the same in all panels. RT patterns are consistent with choice preferences. For EV-sensitive samplers, the peak RT corresponds to ΔEV of ~-50, which is also the steepest portion of the average sigmoid curves characterizing reveal decisions (**Fig. 2a**), but this pattern is not shown by Uncertainty-only or Random participants. Participants with Random and Uncertainty-only strategies had equivalent average RTs.

Supplementary Figure 3 (Fig. S3)

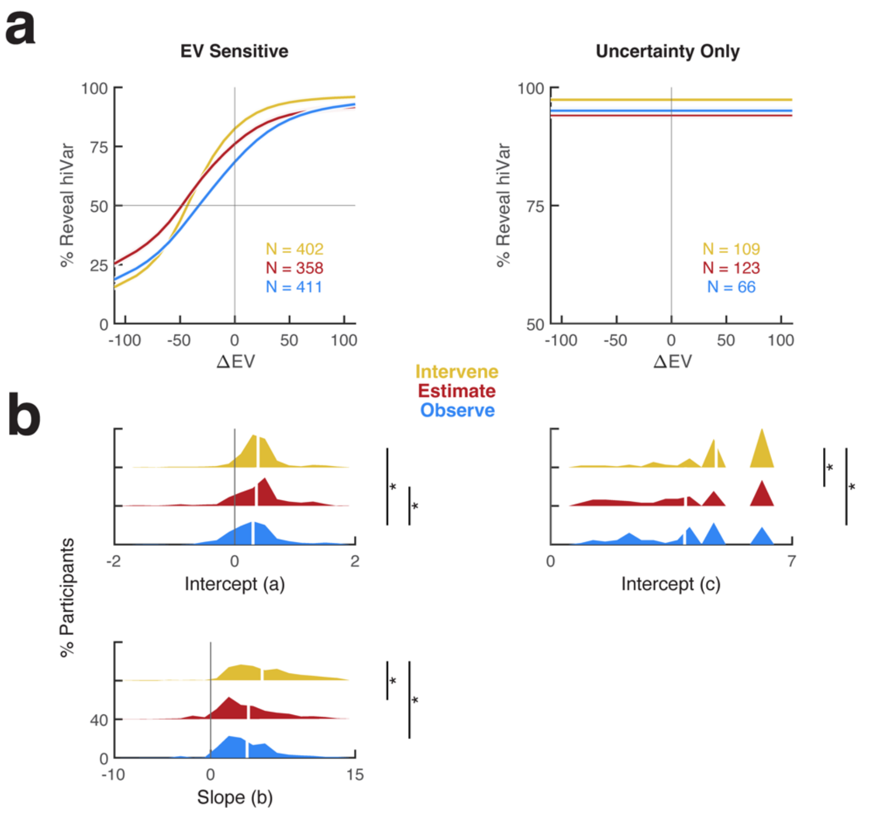

Fig. S3. Unpaired comparisons across blocks (a) Average of fitted decision curves across all participants who had EV sensitive (left) or Uncertainty only (right) sampling in any one block regardless of their sampling in other blocks (N is the number contributing to each curve). **(b) Fit parameters.** Each panel shows the distributions of model parameters for participants in the corresponding columns in **a** (slope and intercept for the left column; intercepts only for the right column). The white lines mark the median of each distribution. Vertical bars and stars show $p < 0.01$ for comparisons across blocks (Wilcoxon signed rank test).

Task effects across all participants (rather than only those who had consistent strategies across the three blocks) confirmed the results in **Fig. 3a,b**.

Instrumental conditions produced higher sensitivity to uncertainty in EV-sensitive samplers (**Fig. S3a**, left). Average values of parameter *a* were 0.30 ± 0.021 in the Observe task, versus 0.36 ± 0.028 in the Estimate task and 0.39 ± 0.019 in the Intervene task, with the latter two values being significantly higher than those on the Observe task (both $p < 0.001$, Mann-Whitney rank-sum test, n = 411, 358 and 402 in, respectively, Observe, Estimate and Intervene tasks). In Uncertainty only participants, parameter *c* was 3.88 ± 0.19 in Observe, 3.90 ± 0.16 in Estimate and 4.79 ± 0.14 in Intervene, with the value in the Intervene task being significantly higher than the Observe task (**Fig. S3b**, right; $p < 0.001$, Mann-Whitney rank-sum test; n = 109 and 66; Estimate versus Observe, $p = 0.994$, Mann-Whitney rank-sum test, n = 123 vs 66).

In addition, sampling was sensitive to the specific instrumental demands, as shown by comparisons between Intervene vs Estimate tasks. In EV-sensitive participants, the average choice function had a steeper slope, suggesting higher EV sensitivity, in Intervene as compared to the Estimate task (**Fig. S3a**, left). Average values of the slope parameter *b* were 3.78 ± 0.14 in Observe, 3.92 ± 0.18 in Estimate and 5.34 ± 0.18 in the Intervene task, indicating significantly higher slopes in the Intervene

vs both the Estimate and Observe tasks (both $p < 0.001$, Mann-Whitney rank-sum tests, n = 411, 358 and 402 participants, in, respectively, Observe, Estimate and Intervene tasks). Uncertainty-only participants had higher uncertainty sensitivity in the Intervene vs Estimate tasks (**Fig. S3b**, right; comparison of parameter c, $p < 0.001$, Mann-Whitney rank-sum, n = 123 vs 109 participants). Together, these findings suggest that the opportunity to alter rather than merely estimate an outcome produced less stochastic and more group-typical sampling. This conclusion is consistent with the fact that, relative to the Estimate task, the Intervene task showed higher R^2 values for fitted choice functions (0.85 ± 0.01 vs 0.72 ± 0.01; $p < 0.001$, Wilcoxon signed rank test, n = 610 participants), a lower incidence of Random strategies (**Fig. 2c;** one-way chi-square test, 16% vs 21%, χ^2 (df = 1) = 4.85, p = 0.027, effect size V = 0.063, n = 610 participants) and more stable choices with less evidence for learning during a block (**Fig. S6c**).

Supplementary Figure 4 (Fig. S4)

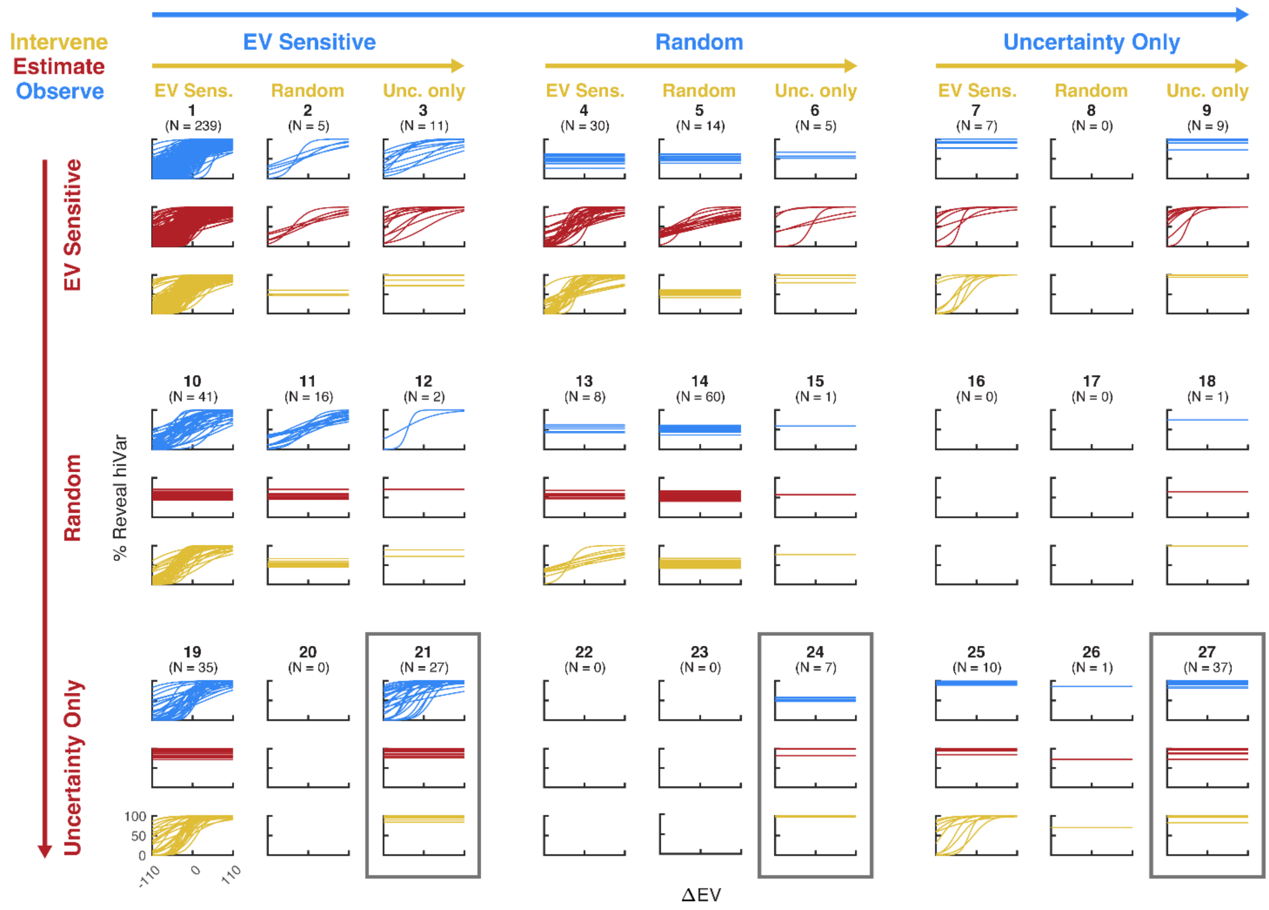

Figure S4. Individual strategy shifts across the whole task. Three-dimensional categorization of individual behavior in the three blocks. Psychometric curves are organized such that the major columns separate by category in Observe (blue), the rows separate by category in Estimate (red), and the minor columns separate by category in Intervene (yellow). Within each of the 27 possible category combinations, individual psychometric curves are plotted for each block, stacked vertically. The numbers show an arbitrary group label (in bold) and the number of participants (in parentheses). Participants who exhibited uncertainty only strategies in the instrumental blocks – groups 21, 24, and 27 – are highlighted with a grey box. Participants with negatively-sloped EV sensitive strategies were excluded to enhance readability. A minority of participants sampled optimally in at least both instrumental tasks (11.6%, groups 21, 24, 27). All other groups show non-normative behavior in at least one instrumental task, and mixed strategies are particularly dominant (39%, group 1).

Supplementary Figure 5 (Fig. S5)

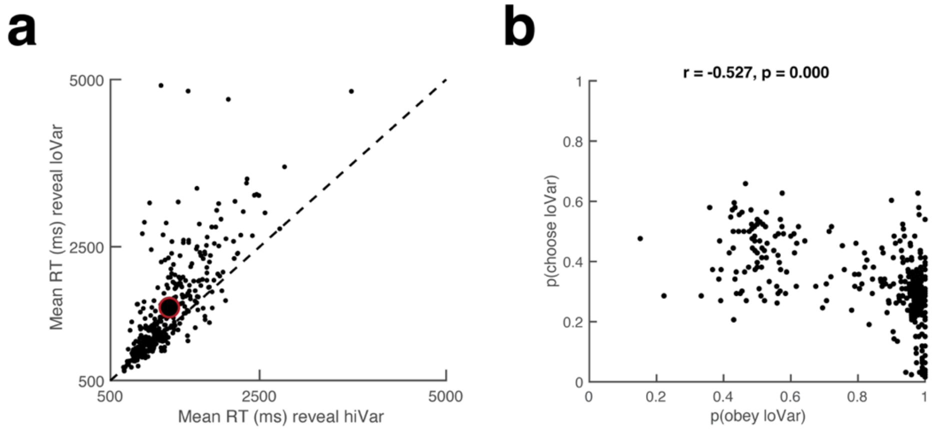

Fig. S5. Sampling and instrumental decision on the Estimate Task. (a) Reaction times (RTs) for the estimation decision in EV-sensitive participants were longer after sampling the loVar relative to the hiVar lottery, consistent with higher estimation uncertainty in the former case. Each point is one participant and the large circle outlined in red indicates the mean (standard error is smaller than the symbol) across all participants (n = 358). The dashed line is the identity line. **(b) Relationship between the sampling and estimations decisions.** Across participants with EV-sensitive strategies in the Estimate block (n = 358), the probability of revealing the loVar lottery is negatively correlated with the probability of estimating congruent with the value revealed from the lottery. The text shows the two-tailed Spearman correlation coefficient and its p-value.

A possible hypothesis as to why EV-sensitive participants frequently sampled the loVar lottery is that they erroneously believed that the draw from this lottery was predictive of the total payoff, potentially by being correlated with the hiVar draw.

This hypothesis makes two predictions for the Estimate task. First, when estimating the sum in this task, participants who held this belief should be just as confident after a loVar or a hiVar observation,. However, contrary to this view, RTs for estimation decisions were significantly longer, suggesting that participants had higher uncertainty after sampling the loVar lottery (**Fig. S5a**; $p < 0.001$; Wilcoxon signed rank test, $n = 358$ participants with EV sensitive strategies in the Estimate task). Second, participants who were more likely to sample the loVar lottery should also be more likely to obey the information from this lottery – i.e., to estimate consistent with a loVar observation, which they believed to be predictive of the total payoff. However, contrary to this view, we found a significant negative correlation, whereby participants who more frequently sampled the loVar lottery were less likely to respond congruently with the draw it revealed (**Fig. S5b**, Spearman's $r = -0.527$, $p < 0.0001$, $n = 358$ participants).

In sum, when EV-sensitive participants sampled the loVar lottery, this did not appear related to the confidence in the estimation decisions they made based on this lottery.

Supplementary Figure 6 (Fig. S6)

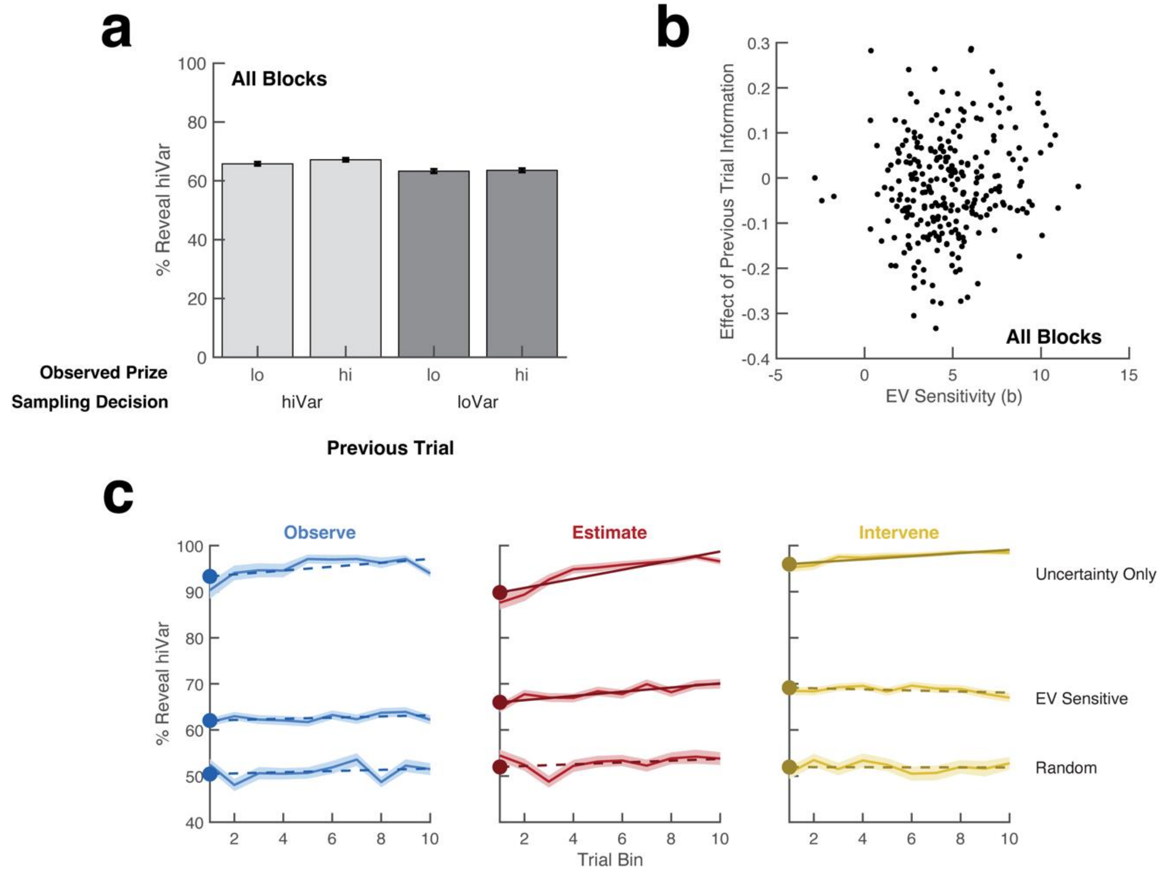

Fig. S6. **Trial by trial learning (a) Influences from revealed information on future sampling.** The probability of sampling the hiVar lottery in trials that followed sampling of a hiVar or loVar lottery (light gray vs dark gray, lower label), separated by whether the observed draw was high or low (two bars within each grayscale level; top labels) for all participants. The bars show mean and standard error across all participants (n = 610) in all blocks. **(b) Relationship between EV sensitivity and the sensitivity to the previous trial information.** For participants who had EV-sensitive strategies in all three blocks, the effect of prior-trial information (see text below) was uncorrelated with parameter b. Each point shows one participant's metrics averaged across blocks. **(c) Learning within blocks.** Sampling behavior as a function of trial bin (mean and standard error across participants). Circles and lines indicate the intercept and slope of linear regression analysis; solid lines show $p < 0.05$ for slope, dashed lines, N.S.

***Win-stay/lose-shift strategy*.** We reasoned that participants may adjust their sampling based on the information on the previous trial. That is, they would assign higher or lower value to the hiVar lottery if on the previous trial they happened to observe a high draw this lottery or a low draw from the loVar lottery. We thus divided trials according to whether the previous trial had sampling of the hiVar or loVar lottery and whether it revealed a low or high draw. While participants more frequently sampled the hiVar lottery if they had sone so on the previous trial, capturing the consistency in sampling behavior (**Fig. S6a**, light gray versus dark gray bars), they were not influenced by whether they observed a high or low draw from that lottery (two bars within each color). A 2-way ANOVA with post-hoc comparisons produced no significant effect of the previous observation or interaction with the sampling decision (n = 610

participants, all $p > 0.05$). We next focused on the 265 participants who had EV sensitive strategies in all blocks and analyzed the correlation between their individual EV sensitivity (parameter b) and the sensitivity to the previous observation (the absolute difference in %reveal hiVar between trials that followed a high versus low observation. We found no significant Spearman's correlations in any block or in the data averaged across blocks (**Fig. S6b**; $n = 265$, all $p > 0.05$).

***Learning during a block*.** To see if participants displayed learning trends, we examined performance as a function of time in each block. We found no change in the percent correct (PC) of the post-sampling decision despite a decline in RT, suggesting that participants became more accustomed to performing the task.

To test for changes in the sampling decision, we fit the %reveal hiVar in 10 consecutive trial bins spanning each block using linear regression via MATLAB's built in linearfit function (**Fig. S6c**). Participants with random strategies showed no significant learning, supporting the view that they were disengaged from the task (**Fig. S6c**, dashed traces; regression $p > 0.05$). However, some participants with non-random behavior showed significant learning trends that had three noteworthy features.

. First, improvements in sampling were found only in instrumental conditions, suggesting that they were based on some aspect of the post-sampling decision (Fig. **S6c**, solid traces, Estimate Uncertainty-only, $p < 0.001$, $n = 123$ participants; Estimate EV sensitive, $p < 0.001$, $n = 358$ participants; Intervene Uncertainty-only, $p < 0.001$, $n = 109$).

Second, the improvements were greater in the Estimate relative to the Intervene blocks. The differences between %reveal hiVar in the first and last bins were greater in the Intervene vs Estimate blocks in Uncertainty-only participants (8.9% ± 1.4% vs 3.3% ± 1.0%, $p = 0.005$ Wilcoxon signed rank test, $n = 109$ and 123 participants, respectively) and, importantly, also in EV-sensitive participants where this could not be due to ceiling effects (5.2% ± 1.5% vs 1.0% ± 1.1%, $p < 0.001$, Mann-Whitney rank-sum test, $n = 402$ vs 358 participants, respectively).

Third, in both Intervene and Estimate blocks, learning was stronger in Uncertainty-only relative to EV-sensitive participants, despite the fact that the former had more efficient sampling to begin with (and might have been subject to ceiling effects). As detailed above, Uncertainty-only participants showed significant learning in both blocks while EV sensitive participants did so only in the Estimate block (**Fig S6c**). At the individual level, significant learning was shown by 46% of Uncertainty-only participants but only 13% of EV-sensitive participants, a difference that was significant in individual blocks (one-way chi-square test in the Estimate task, $\chi^2(df = 1) = 62.49$; $p < 0.001$, effect size $V = 0.50$; Intervene: χ^2 $(df = 1) = 55.66$, $p < 0.001$, effect size $V = 0.51$).

Together, the findings suggest that participants adjusted their sampling by monitoring the instrumental decision but this was gated by their initial understanding of the efficient strategy.

Supplementary Figure 7 (Fig. S7)

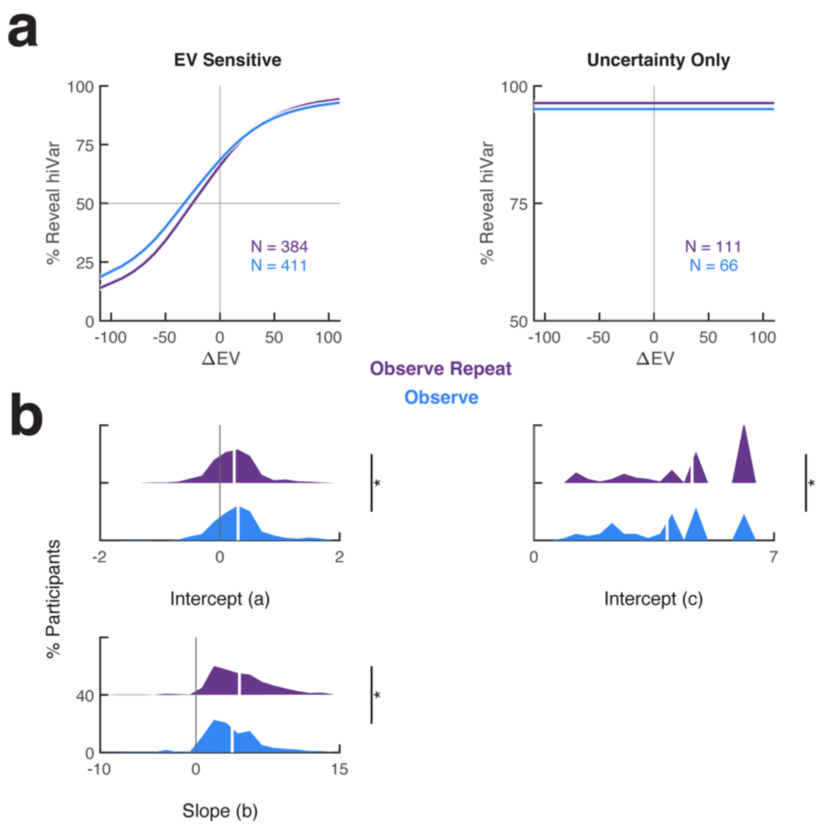

c

Repeat EV Sensitive	Repeat Uncertainty Only
82.5% (339/411)	85% (56/66)

Fig. S7. First and repeated non-instrumental block. (a) Average, model-fit decision curves across all participants who had EV sensitive (left) or Uncertainty only (right) sampling in the respective Observe block (n = 384, 411, 111 and 66 as shown in the panels). **(b) Fit parameters.** Each panel shows the distributions of model parameters for participants in the corresponding columns in **a**. The white lines mark the median of each distribution. Vertical bars and stars show $p < 0.01$ for comparisons across blocks (Wilcoxon signed rank tests). **(c) Consistency.** Fraction of participants who, conditional on having an EV-sensitive or Uncertainty-only strategy in the first Observe block, repeated the same strategy in the final block.

To test if exposure to instrumental conditions affected non-instrumental demand, we compared the first and last Observe blocks. While uncertainty only participants showed a small increase in efficient sampling in the last block (**Fig. S7a,b** right; parameter *c*: 4.61 ± 0.16 vs 3.88 ± 0.19, $p = 0.002$, Mann-Whitney rank-sum test, $n = 111$ and 65, respectively), participants with EV sensitive strategies showed significantly lower sensitivity to uncertainty and higher sensitivity to EV (**Fig. S7a,b** left; intercept, *a*, 0.24 ± 0.02 vs 0.30 ± 0.0, $p = 0.043$; slope, *b*: 4.54 ± 0.16 vs 3.78 ± 0.14 $p < 0.001$; Mann-Whitney rank-sum tests, $n = 384$ vs 411). Across all participants, %reveal hiVar was not significantly different in the first versus last Observe blocks (63.60 ± 16.94% vs 65.09 ± 19.48%; $p = 0.353$; Wilcoxon signed rank test, $n = 610$). Moreover, we found no evidence that participants preferentially repeated Uncertainty-only strategies across blocks (**Fig. S7c**). Overall, we found no credible evidence that participants adopted more efficient non-instrumental information demand after experiencing instrumental conditions.
