# Supplementary material for: Inefficient prioritization of task-relevant attributes during instrumental information demand
Source: Nat Commun. 2023 Jun 1;14:3174. doi: 10.1038/s41467-023-38821-x (PMC10235048; doi:10.1038/s41467-023-38821-x)
Supplement: Supplementary file 2 — Reporting Summary [file 41467_2023_38821_MOESM2_ESM.pdf]

Corresponding author(s): Double-anonymous peer review submissions: write DAPR and your manuscript number here instead of author names. Professor Jacqueline Gottlieb

Last updated by author(s): YY 05/05/2023

Reporting Summary

Nature Portfolio wishes to improve the reproducibility of the work that we publish. This form provides structure for consistency and transparency in reporting. For further information on Nature Portfolio policies, see our Editorial Policies and the Editorial Policy Checklist.

Statistics

For all statistical analyses, confirm that the following items are present in the figure legend, table legend, main text, or Methods section.

n/a	Confirmed	
☐	☒	The exact sample size (*n*) for each experimental group/condition, given as a discrete number and unit of measurement
☐	☒	A statement on whether measurements were taken from distinct samples or whether the same sample was measured repeatedly
☐	☒	The statistical test(s) used AND whether they are one- or two-sided<br>*Only common tests should be described solely by name; describe more complex techniques in the Methods section.*
☐	☒	A description of all covariates tested
☐	☒	A description of any assumptions or corrections, such as tests of normality and adjustment for multiple comparisons
☐	☒	A full description of the statistical parameters including central tendency (e.g. means) or other basic estimates (e.g. regression coefficient) AND variation (e.g. standard deviation) or associated estimates of uncertainty (e.g. confidence intervals)
☐	☒	For null hypothesis testing, the test statistic (e.g. *F*, *t*, *r*) with confidence intervals, effect sizes, degrees of freedom and *P* value noted<br>*Give P values as exact values whenever suitable.*
☐	☒	For Bayesian analysis, information on the choice of priors and Markov chain Monte Carlo settings
☒	☐	For hierarchical and complex designs, identification of the appropriate level for tests and full reporting of outcomes
☐	☒	Estimates of effect sizes (e.g. Cohen's *d*, Pearson's *r*), indicating how they were calculated

Our web collection on statistics for biologists contains articles on many of the points above.

Software and code

Policy information about availability of computer code

Data collection	Data were collected using custom software (Haratki LLC) implemented on Amazon Mechanical Yurk
Data analysis	Data were analyzed using Matlab version 2010a

For manuscripts utilizing custom algorithms or software that are central to the research but not yet described in published literature, software must be made available to editors and reviewers. We strongly encourage code deposition in a community repository (e.g. GitHub). See the Nature Portfolio guidelines for submitting code & software for further information.

Data

Policy information about availability of data

All manuscripts must include a data availability statement. This statement should provide the following information, where applicable:

- Accession codes, unique identifiers, or web links for publicly available datasets
- A description of any restrictions on data availability
- For clinical datasets or third party data, please ensure that the statement adheres to our policy

Source data are provided with this paper. The processed lottery and questionnaire data generated in this study have been deposited in the EBRAINS Database. Access can be obtained through https://doi.org/10.25493/ZQZM-PPS. The raw lottery and questionnaire data are protected and are not available due to data privacy laws.

Research involving human participants, their data, or biological material

Policy information about studies with human participants or human data. See also policy information about sex, gender (identity/presentation), and sexual orientation and race, ethnicity and racism.

Reporting on sex and gender	We collected demographic data through a brief optional questionnaire presented on Amazon Turk. OF the 550 participants who chose to provide the data, 45% self identified as women and 55% men (0% identified as "other").<br>We found no credible evidence that this self identification affected informations sampling decisions (Fig. 4), confirming previous studies on this topic.
Reporting on race, ethnicity, or other socially relevant groupings	We collected demographic data through a brief optional questionnaire. In addition to gender, participants were asked about their age (categories: 18-30, 31-35, 36-55 and 56-75 year old), and highest degree achieved (high school, college, post-graduate, vocational). No other questions were asked.
Population characteristics	Demographic data (collected from 550 participants), shows that their ages ranged between 18-75 years old (median category, 31-35 year old), 45% were women (55% men, 0% other), and a majority completed college (58%) or a post graduate degree (24%) with the remaining having completed only high school (17%) or a vocational school (1%).
Recruitment	Participants were recruited through the online platform Amazon Mechanical Turk . To ensure quality data, we limited enrollment to participants who were (self-declared) adults over 18 in the United States, and who were verified as having completed more than 100 previously approved Amazon Turk tasks with an approval rate of over 80%. No other criteria were considered because (a) they are difficult to evaluate on Amazon Turk, and (b) we have no prior hypotheses we wished to test with respect to information sampling decisions.
Ethics oversight	the Institutional Review Board of Columbia University

Note that full information on the approval of the study protocol must also be provided in the manuscript.

Field-specific reporting

Please select the one below that is the best fit for your research. If you are not sure, read the appropriate sections before making your selection.

- [ ] Life sciences
- [x] Behavioural & social sciences
- [ ] Ecological, evolutionary & environmental sciences

For a reference copy of the document with all sections, see nature.com/documents/nr-reporting-summary-flat.pdf

Behavioural & social sciences study design

All studies must disclose on these points even when the disclosure is negative.

Study description	Participants completed cognitive tasks and personality questionnaires on Amazon Mechanical Turk. Data were analyzed with quantitative methods.
Research sample	Participants were recruited online using Amazon Mechanical Turk. This provides a wider sample relative to in-person studies that typically focus on college students or participants from a single community.
Sampling strategy	Sampling was only determined by participation on Amazon Mechanical Turk. To ensure the broadest possible sampling we only used restrictions that ensured quality data: we limited enrollment to participants who were (self-declared) adults over 18 in the United States, and who were verified as having completed more than 100 previously approved Amazon Turk tasks with an approval rate of over 80%.
Data collection	All data were collected on Amazon Mechanical Turk. No participant was tested in-person. Participants performed cognitive tasks and filled out questionnaires on their computer, entered responses through a keyboard and the data were electronically saved.
Timing	Data were collected between October 2020 and January 2021
Data exclusions	No data were excluded from the analyses.
Non-participation	Participants voluntarily chose to participate. No participant dropped out.
Randomization	Participants were not allocated to treatment groups.

Reporting for specific materials, systems and methods

We require information from authors about some types of materials, experimental systems and methods used in many studies. Here, indicate whether each material, system or method listed is relevant to your study. If you are not sure if a list item applies to your research, read the appropriate section before selecting a response.

Materials & experimental systems

n/a	Involved in the study
☒	☐ Antibodies
☒	☐ Eukaryotic cell lines
☒	☐ Palaeontology and archaeology
☒	☐ Animals and other organisms
☒	☐ Clinical data
☒	☐ Dual use research of concern
☒	☐ Plants

Methods

n/a	Involved in the study
☒	☐ ChIP-seq
☒	☐ Flow cytometry
☒	☐ MRI-based neuroimaging
